# Label-Free SERS Discrimination and In Situ Analysis of Life Cycle in *Escherichia coli* and *Staphylococcus epidermidis*

**DOI:** 10.3390/bios8040131

**Published:** 2018-12-15

**Authors:** Niccolò Paccotti, Francesco Boschetto, Satoshi Horiguchi, Elia Marin, Alessandro Chiadò, Chiara Novara, Francesco Geobaldo, Fabrizio Giorgis, Giuseppe Pezzotti

**Affiliations:** 1 Department of Applied Science and Technology, Politecnico di Torino, C.so Duca degli Abruzzi 24, 10129 Turin, Italy; niccolo.paccotti@polito.it (N.P.); chiara.novara@polito.it (C.N.); francesco.geobaldo@polito.it (F.G.); 2 Ceramic Physics Laboratory, Kyoto Institute of Technology, Kyoto 606-8585, Japan; boschetto.cesc@gmail.com (F.B.); elia-marin@kit.ac.jp (E.M.); pezzotti@kit.ac.jp (G.P.); 3 Department of Immunology, Kyoto Prefectural University of Medicine, Kyoto 602-0841, Japan; shorigu@koto.kpu-m.ac.jp; 4 Department of Dental Medicine, Kyoto Prefectural University of Medicine, Kyoto 602-0841, Japan; 5 Department of Orthopedic Surgery, Tokyo Medical University, Tokyo 160-8402, Japan; 6 The Center for Advanced Medical Engineering and Informatics, Osaka University, Osaka 565-0871, Japan

**Keywords:** SERS, metal-dielectric nanostructures, *E. coli*, *S. epidermidis*, biofilm

## Abstract

Surface enhanced Raman spectroscopy (SERS) has been proven suitable for identifying and characterizing different bacterial species, and to fully understand the chemically driven metabolic variations that occur during their evolution. In this study, SERS was exploited to identify the cellular composition of Gram-positive and Gram-negative bacteria by using mesoporous silicon-based substrates decorated with silver nanoparticles. The main differences between the investigated bacterial strains reside in the structure of the cell walls and plasmatic membranes, as well as their biofilm matrix, as clearly noticed in the corresponding SERS spectrum. A complete characterization of the spectra was provided in order to understand the contribution of each vibrational signal collected from the bacterial culture at different times, allowing the analysis of the bacterial populations after 12, 24, and 48 h. The results show clear features in terms of vibrational bands in line with the bacterial growth curve, including an increasing intensity of the signals during the first 24 h and their subsequent decrease in the late stationary phase after 48 h of culture. The evolution of the bacterial culture was also confirmed by fluorescence microscope images.

## 1. Introduction

The development of a rapid, reproducible, and sensitive method to detect pathogens is important in several different areas of our everyday life, including food and water safety, as well as in a number of biomedical applications [1,2,3,4]. In the last decades, molecular vibrational methods, namely, infrared and Raman spectroscopies, have attracted significant interests in the investigations of biological environments and bacteria characterizations [5,6]. Surface enhanced Raman spectroscopy (SERS), as an ultra-sensitive and non-destructive technique, emerged as one of the most promising approaches for biological analysis [7,8,9,10]. Thanks to the huge enhancement of the Raman scattering signal, this technique enables the detection of extremely low concentrations of molecules adsorbed onto the surface of plasmonic substrates [11,12]. The main contribution to the SERS effect is because of the electromagnetic amplification arising from the localized surface plasmons (LSP) held up by the nanostructures. Several techniques have so far been developed for manufacturing highly efficient and reproducible solid SERS substrates [13,14,15]. However, despite continuous improvements, some drawbacks concerning the high costs of fabrication, the process complexity, and the related reliability still limit their use. To address these complications, silvered porous silicon (pSi) substrates have been developed as solid SERS platforms [16]. These substrates provide both good sensitivity and reproducibility, while taking advantage of characteristics such as low fabrication cost and high application flexibility [4,11]. Moreover, these substrates have been recently integrated with a thin membrane of polydimethylsiloxane (PDMS) hosting the porous silicon layer decorated with silver nanoparticles (Ag-NPs) [4,17].

Bacteria are prokaryotic organisms with dimensions of about a few micrometers. Their cells are surrounded by the cytoplasmic membrane, which is covered by an outer layer whose structure and composition differs among different bacterial strains, and forms the cell envelope [5]. The main classification of the bacteria falls into the division between Gram-positive and Gram-negative species, based on Gram staining differentiation [18]. This differentiation exploits the composition of the cell membranes, which is distinctive for each of the two categories of bacteria [5,19]. The envelope of the Gram-negative bacteria is composed by an outer membrane, which is rich in lipopolysaccharides and phospholipids, and by an inner cytoplasmic membrane. A thin layer of peptidoglycan separates these two membranes. Conversely, the Gram-positive bacteria cell wall consists of a cytoplasmic membrane covered by a thick layer of peptidoglycans, while the outer phospholipid envelope is missing [5,20]. These differences, strongly affecting the well-known susceptibility to antibiotics of these two groups of bacteria [18], yield different Raman spectral features that can be used to selectively differentiate these bacterial populations.

In this work, the PDMS-supported silver-coated pSi substrates have been used to provide a simple and reliable analytical protocol to discriminate Gram-positive and Gram-negative bacterial strains, minimally affecting the bacterial metabolism. *Staphylococcus epidermidis* and *Escherichia coli* were selected as the representative strains belonging to these two groups. Both of the selected strains have been extensively studied using Raman spectroscopy and SERS, typically using substrates based on colloidal solutions of Au or Ag-NPs [21,22,23,24]. The strains used for the tests are not pathogenic, reducing any risk for the operator, even if both of the bacteria can be seen as opportunistic pathogens, among the most frequent in nosocomial infections [25,26].

The proposed method represents a label-free approach, for which the following is intended: (i) to resolve the full set of vibrational modes from the molecular species present in both *E. coli* and *S. epidermidis* strains, and (ii) to exploit such differences to discriminate between Gram-negative and Gram-positive species. Furthermore, in this study, we attempted to deconvolve and give assignments to all of the SERS bands, thus evaluating the evolution of each component throughout the complete growth curve of the different bacteria based on an in-situ vibrational spectroscopic analysis. Fluorescence microscopy micrographs of the living and dead cells were taken at different times of culture, so as to support and confirm the results obtained by the SERS analysis.

## 2. Materials and Methods

### 2.1. Fabrication of SERS Substrates

The SERS nanostructures were synthesized starting from a porous silicon layer (pSi), prepared by electrochemical etching (102 mA/cm^2^, 35 s) of boron doped silicon wafers (34–40 mΩ-cm resistivity), in a 20:20:60 water, ethanol, and hydrofluoric acid (HF) mixture. A second anodization took place with a current density of 3.7 mA/cm^2^ for 40 s in a low concentrated HF ethanol solution to weaken the pore walls at the silicon/pSi interface. The porous silicon layer was transferred on a partially cross-linked PDMS slice with a 10:1 ratio of oligomer and curing agent. The Ag-NPs were synthesized by dipping the pSi/PDMS substrates for 15 s at 20 °C in a 10^−2^ M silver nitrate (AgNO_3_) aqueous solution, to which a 0.0006% *v*/*v* of HF was added. The samples were analyzed by field emission scanning electron microscopy (FESEM), by acquiring secondary electron contrast images with 5 keV electrons using an in-lens detector of a Zeiss SUPRA 40 microscope (Zeiss SMT, Oberkochen, Germany). The samples were previously covered with a copper grid anchored to the FESEM stub so as to dissipate the charge loading. The detailed morphological characterization and the evaluation of the SERS performances of the SERS substrates were previously reported [11].

### 2.2. Bacterial Culture and Viability Test

**Figure 1:**
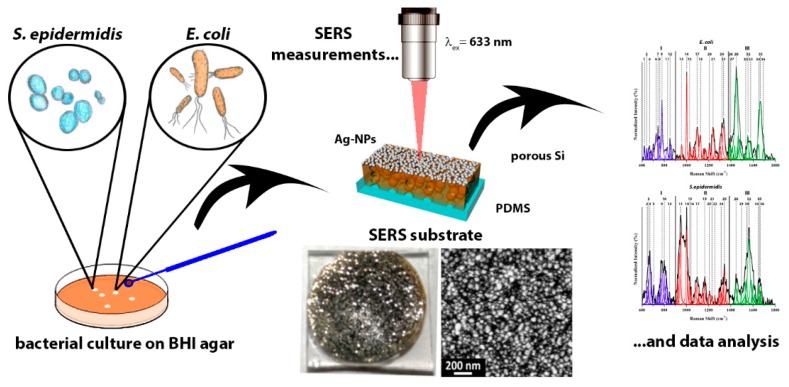
Scheme of the surface enhanced Raman spectroscopy (SERS) analysis of *E. coli* and *S. epidermidis* grown on brain heart infusion (BHI) agar and collected after different times of culture.

Bacterial cells of both *S. epidermidis* (14990 ^®^ATCC^TM^) and *E. coli* (25922 ^®^ATCC^TM^) were cultured at the Kyoto Prefectural University of Medicine on a brain heart infusion (BHI) agar medium (Nissui, Tokyo, Japan). The initial concentration of bacteria was assessed by measuring the optical density (OD) at 650 nm with a E-MAX microplate reader (Molecular Devices, Tokyo, Japan), and the inoculum was obtained by diluting the bacterial suspension to 1.0 × 10^8^ CFU/mL, using a phosphate buffer saline (PBS) solution. Finally, 100 µL of bacteria solutions were plated onto a petri dish containing BHI agar. The plates were incubated at 37 °C under aerobic conditions for three selected times, namely, 12, 24, and 48 h. These bacterial culture on the agar plates were used for the SERS analysis, as described below (Figure 1). In order to evaluate the microbial viability, the Microbial Viability Assay Kit-WST8 (Dojindo Laboratories Co., Ltd., Kumamoto, Japan) was used, by following the manufacturer technical manual. Briefly, the biofilm grown on the agar plates was resuspended in PBS, whose concentration of bacteria was estimated by measuring the OD at 650 nm. The suspension was eventually diluted to 1.0 × 10^8^ CFU/mL, and then 190 µL were incubated with the WST8 reagent (2-(2-methoxy-4-nitrophenyl)-3-(4-nitrophenyl)-5-(2,4-disulfophenyl)-2H-tetrazolium) for 30 min at 37 °C. Afterwards, the OD at 490 nm was measured with the E-MAX microplate reader. Three replicates have been used to calculate the mean values, and the error bars represent the standard deviations of these measurements.

### 2.3. SERS Analysis

The bacteria cultivated for different times were transferred on the SERS substrates, which were previously sterilized trough UV exposure for 24 h (15 W), directly from the agar plate and immediately before the SERS experiments. The spectra were collected using a confocal Raman microscope (LabRAM HR800; Horiba/Jobin-Yvon, Kyoto, Japan), with a 633 nm laser excitation using a 100 × objective. The laser beam was focused at the interface between the Ag-NPs and the bacterial biofilm. The average spectra were calculated from 10 different measurements acquired at different spots on the samples at each time of cultivation. All of the spectra were normalized and processed using a commercial software (LabSpec 5.0, Horiba/Jobin-Yvon, Kyoto, Japan and Origin 8.5, OriginLab Co., Northampton, MA, USA).

### 2.4. Fluorescence Microscope Images

The fluorescence microscope images of bacterial samples collected after 12, 24, and, 48 h of culture were acquired for both of the bacterial strains. The cells were previously stained with propidium iodide (PI), 5(6)-carboxyfluorescein diacetate (CFDA), and 4′,6-diamidino-2-phenyilindole (DAPI), which colored the nucleic acids (dead bacteria; red), live bacteria (green), and nucleic acids (blue), respectively, by following the manufacturer’s protocol (Bacstain, Dojindo Laboratories Co., Ltd., Kumamoto, Japan). A constant magnification of 10 × was chosen for all of the samples. In order to estimate the amount of viable and dead bacteria, the images related to PI and CFDA were analyzed by means of ImageJ 1.50e, and the percentage of coverage was obtained from each single channel (red and green). Three replicates were used to calculate the mean values, and the error bars represent the standard deviations of these measurements.

## 3. Results and Discussions

### 3.1. Labelling SERS Spectrum of E. coli and S. epidermidis

**Figure 2:**
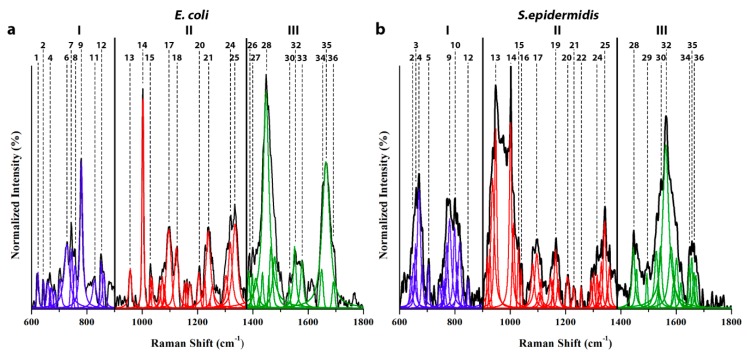
SERS spectra of *E. coli* (**a**) and *S. epidermidis* (**b**) in the 600 to 1800 cm^−1^ range, collected after 24 h of culturing. The spectrum was divided into three distinct zones (labeled zones I to III), while the band frequencies were labeled according to Table 1.

In order to fully resolve the vibrational modes belonging to the molecular species, both *E. coli* and *S. epidermidis* were cultured on agar plates, and samples of the bacteria were directly collected from the plates. Figure 2 shows the average SERS spectra collected from *S. epidermidis* and *E. coli* living bacteria after 24 h of culture on agar plates, after their transfer on the Ag-coated pSi-PDMS membranes, according to the scheme in Figure 1. This cultivation time was selected in order to obtain the maximum concentration of living bacterial cells, which, according to the literature, is reached during the stationary phase [27,28]. A comparison of the SERS and normal Raman spectrum of *E. coli* is also presented in the Supporting Material, Figure S1, to highlight the richer information content provided by the SERS analysis.

The spectra, which are displayed in the energy range between 600 and 1800 cm^−1^, were divided into three main regions, henceforth referred to as low (600–900 cm^−1^), middle (900–1380 cm^−1^), and high (1380–1800 cm^−1^) frequency regions. The vibrational modes of each of the 36 deconvoluted bands were assigned according to the literature (Table 1). Comparing the two spectra, many differences could be observed both as variations in intensity and/or spectral shifts of the relative Raman bands. All of those spectroscopic features correspond to compositional differences in the molecular structure of the two bacterial strains, including their biofilm matrix, as discussed below.

The main difference in Zone I (low frequencies, 600–900 cm^−1^) is related to the band located at around 665~670 cm^−1^ (labelled Band 1 in Figure 2a,b, and in Table 1). Such a band has been associated with the presence of *N*-acetylglucosamine (NAG) [27,29], which, along with *N*-acetylmuramic acid (NAM), is one of the main components of the linear chains of amino-sugars that compose the peptidoglycan layer. The thickness of this shell is one of the major features used to distinguish between Gram-positive and Gram-negative bacteria. The cell wall of the Gram-positive *S. epidermidis* consists of a thick layer of peptidoglycans [27], which surrounds the cytoplasmic membrane, while in the membrane of the Gram-negative *E. coli*, this envelope is much thinner. Moreover, *S. epidermidis* is known to be able to produce biofilms composed of aggregated cells and extracellular polymeric substances (EPS), mainly made of polysaccharide intercellular adhesin (PIA), a linear exopolysaccharide consisting of β(1,6)-linked *N*-acetyl-glucosamine residues [30]. Instead, the membrane of Gram-negative bacteria displays an additional layer mostly made of phospholipids (as discussed later). A clear fingerprint of the peptidoglycan membrane structure is seen as a difference in the intensity of the NAG related Band 3 (and other sub-bands in its spectral neighbourhood), which is stronger in *S. epidermidis* (Figure 2b) than in *E. coli* (Figure 2a).

Additional bands in Zone I are related to the twisting mode of phenylalanine at 621 cm^−1^ [19] (only in *E. coli*), and to the vibrational modes of the nitrogenous bases of nucleic acids, located at ~650 cm^−1^, 660 cm^−1^ (Band 3; only in *S. epidermidis*) [17,19], 725 cm^−1^ (Band 6) [4,17,31,32], 780 cm^−1^ (Band 9) [17,27,29,31], 800 cm^−1^ (Band 10; only in *S. epidermidis*) [27,29], and ~850 cm^−1^ (Band 12) [17,19]. In particular, Band 6 at 725 cm^−1^ belongs to the ring breathing of adenine [4,19,31,32], which can be ascribed to the presence of extracellular DNA (eDNA), or to other common adenine-containing molecules, such as RNA, FAD, NAD, AMP, ADP, or ATP, which are critical molecules for the biochemical processes of cells [33]. From the comparison between the two spectra, it can be noticed that the DNA related bands (Band 6–9) are less intense in the *S. epidermidis* spectrum. Such differences can be due to the different amount of eDNA in the extracellular matrix, which was found to be a minor component in the biofilm produced by *S. epidermidis* [34].

The presence of eDNA is also justified by the signal at 650 cm^−1^ (Band 2), which can be ascribed to guanine, to the one around 780 cm^−1^ (Band 9), which has been assigned to the ring breathing of cytosine and thymine, and to the one at 848~851 cm^−1^, assigned to the vibrational modes of thymine (Band 12). The vibrational features of the nitrogenous bases, and in particular, the signal located at 780 cm^−1^, could have two possible explanations, as follows: a first hypothesis takes into account the presence of intracellular DNA, while another interpretation refers once more to the presence of eDNA, which can be found in the biofilm matrix [24]. Comparing the results obtained in this work with those shown in other studies [19,32,35], the explanation involving the eDNA is seen as the most plausible, taking into account that the eDNA could better interact, rather than the intracellular DNA, with the Ag NP on the surface of the substrates. It is worth noting that the majority of previous works take advantage of metal NPs (Au and Ag) dispersed in water as a colloidal solution, applying a treatment protocol of the bacteria samples that aims to remove the extracellular components of the biofilm matrix [19,31,32,36]. For this reason, the SERS characterization of *E. coli*, carried out by Lemma et al., revealed that this band is completely missing [19], whereas in the results displayed by Witkowska et al., it has a really low intensity [31]. The lack of this vibrational feature could be due to the removal of the biofilm matrix, supporting the eDNA hypothesis. Other strong differences with respect to the literature are related to the intensity of the adenine ring breathing (Band 6), which usually dominates this region of the spectra, whereas in this work, it is overlooked by the signal originated from the ring breathing of cytosine and thymine (Band 9, and only in *S. epidermidis* Band 10).

Band 7, located at 744 cm^−1^, was attributed to the B_1g_ vibration of the heme group of cytochrome c, a small hemeprotein that plays an essential role in the electron transport chain [35,37,38,39,40]. A second fingerprint evidencing the presence of the cytochrome c could be Band 18, located at 1124 cm^−1^ (with-in Zone II); however, the literature also reports several alternative assignments concerning this vibrational mode, namely: backbone C–C skeletal modes of protein [36,41], C–C vibration of unsaturated fatty acids [36], and modes of phosphate groups belonging to nucleic acids [19,36]. These last assignments can be more reasonable, especially for *E. coli*, because they fit with the presence of lipopolysaccharides (LPS) on the surface of the Gram-negative bacteria [42]. The *E. coli* spectrum also shows the presence of a tryptophan band at 755 cm^−1^ (Band 8) [37,43], and a weak signal at 826 cm^−1^, which has been attributed to the vibrational modes of the O–P–O phosphate groups [19,44]. Once again, the signals of the phosphate groups can be due to the presence of eDNA or to the LPS that is found only on the *E. coli* outer membrane.

Zone II (medium frequencies, 900–1380 cm^−1^) is dominated by the peak of phenylalanine at 1002 cm^−1^ (Band 14), which is an amino acid playing a key role in the formation of proteins. Furthermore, the abundance of this compound can be used as a marker for cell viability [45]. The sharp character of this band makes it suitable to normalize the overall spectrum. Additional bands related to the presence of phenylalanine are located at around 1030 cm^−1^ and 1204 cm^−1^ (Bands 15 and 20, respectively) [19,27,29,31,37]. The characteristic features of the proteins are displayed by the amide I, II, and III bands, which arise from the bond vibrations in the peptide units. Each of these vibrational modes lies in a distinct region of the spectrum, providing a description of the secondary structure of the peptides [46]. The amide III vibrational modes range from ≅1150 cm^−1^ to ≅1350 cm^−1^. In particular, intense bands related to amide III are located at 1165 cm^−1^ and 1230–1241 cm^−1^ (Bands 19 and 21, respectively) [19,29], while the twisting of CH_2_ and CH_3_ (Band 22) are located at about 1335 cm^−1^ (*E. coli*) and 1342 cm^−1^ (*S. epidermidis*) [29,37]. An additional band related to the vibrations of the methyl group involved in the α-helix structures is located around 950 cm^−1^ (Band 13) [19]. It is worth noting that, according to the literature, the contribution to the signal of Band 21 is due both to amide III and to the phosphate groups’ vibrations [19,29]. These assumptions match perfectly with the difference between the spectra of the two bacterial strains, which show a more intense signal in the *E. coli* spectrum with respect to the *S. epidermidis* one; such diversity could be due to the contributions of the phosphate groups located in the LPS present on the outer membrane of *E. coli* and on the membrane itself [42], whereas in *S. epidermidis*, the only contribution to this vibrational feature is due to the presence of teichoic acids on the external layer of the peptidoglycan wall [5,47]. Indeed, an additional band due to the vibration of the phosphate groups can be located at ≅1096 cm^−1^, (Bands 17) [19,44], even if it can be assigned both to the phospholipids and nucleic acids. Moreover, the presence of nucleic acids is pointed out by the signal related to the vibration of guanine, located at around 1319 cm^−1^ (Band 24) [19,29]. Even though this region does not show big differences compared to the literature, it is worth noting that a comprehensive and detailed characterization of the spectra is provided showing an enhanced sensitivity, with respect to previous works [19,29,31,48].

Zone III (high frequencies; 1380–1800 cm^−1^) is characterized by the vibrational activity of proteins (amide I and amide II) and fatty acids, which constitute the three main signals. Amide I bands arise mostly from C=O stretching vibrations combined with some contributions from the N–H bending and from the C–C–N deformation [46]. In particular, the carbonyl and amino groups are involved in the hydrogen bonds between different peptide residues inside the protein framework [46]. The bands relative to amide I usually appear in a wide range of frequencies, fluctuating from 1640 cm^−1^ to 1694 cm^−1^, as shown in Figure 2a,b. It can be noted that these bands overlap each other, making it difficult to isolate their individual contributions; moreover, a small contribution in the early amide I region originating from the vibrations of the carbonyl group from cytosine and thymine has to be taken into account [17]. Because of these characteristics, despite a univocal interpretation of the spectrum could hardly be obtained, we attempted to identify the contribution of each vibrational mode, taking into account the previous literature and the different structures of the two microorganisms (Table 1). As this set of bands does not overlap with any other vibrational modes of protein, the literature usually refers to amide I as a marker for the interpretation of the protein secondary structure [48]. Nevertheless, in the SERS spectra of peptides and proteins, the amide I band is often suppressed. A possible explanation is provided by Kurouski et al., who show how the intensity of the SERS signal is influenced by the nature of the amino acids constituting the peptides chains [48,49]. The analysis of several amino acids demonstrates that the presence and the intensity of the amide I band is strongly affected by the proximity to the metal surface, which could depend on the length of the amino acid side chains. Usually, peptide sequences with short side chains, such as –H and –CH_3_, have been proven to show a more intense amide I band, whereas the spectra of the amino acids sequences with longer side chains, like the aromatics groups, display a lack of this vibrational feature [48].

Comparing the regions belonging to the amide I in the spectra shown in Figure 2a,b, it can be seen that the *E. coli* spectrum shows a more intense set of bands with respect to the *S. epidermidis* one. Although it is hard to estimate the contribution of the side chains of the amino acids, this difference could be related to a more substantial presence of proteins, such as porins and other outer membrane proteins (OMPs), through which molecules can diffuse inside or outside the outer layer of the Gram-negative bacteria cell walls [5]. These transmembrane protein channels are mainly composed of hydrophilic amino acids with short side chains [50], which could lead to a more intense signal of the amide I vibrational features in the *E. coli* spectrum (Figure 2a). Other OMPs can have membrane proteins involved in essential functions, such as cell adhesion, cell signaling, or waste export, or even virulence factors (e.g., nutrient scavenging or evasion of host defense mechanisms) [51]. In particular, *E. coli* express different adhesion proteins and protein-based organelles, such as fimbriae, curli, and conjugative pili, when the cells are involved in biofilms [52]. *S. epidermidis*, instead, have a lower amount of proteins in the outer layer of the membrane, if compared to *E. coli*, as it can be seen by the less intense signal of the amide I bands in the spectrum (Figure 2b, Bands 34–36). Indeed, the surface of staphylococci is mainly made of PIA, a homoglycan composed of β-1,6-linked *N*-acetylglucosamine residues and another polysaccharide, referred to as 20-kDa polysaccharide (20-kDaPS), composed of glucose and partially sulfated NAG, which constitute the EPS of the extracellular biofilm matrix, and a small content of proteins, such as autolysins [47,53]. In *S. epidermidis*, these autolysins are responsible for the generation of the eDNA [34]. Such results are supported by the data obtained by Neugebauer et al. [29], in whose work the amide I vibrational modes are absent or strongly suppressed.

The amide II bands consist of NH in-plane bending combined with CN/CC stretching and CO in-plane bending vibrations, typically occurring around 1550 cm^−1^. As it can be noticed by comparing the *E. coli* and *S. epidermidis* spectra in Zone III, several differences appear, especially concerning the vibrational modes related to the presence of phospholipids, at about 1450 cm^−1^ (Band 28) [19,29], and the bands assigned to the amide II of the proteins, located around 1553 cm^−1^ (Band 32) [19,29]. As anticipated above, the reason for such strong differences lies in the composition of the membranes of *E. coli* and *S. epidermidis*, in particular, in their external layer. Gram-negative bacteria display an additional membrane mostly made of phospholipids, which is completely missing in the Gram-positive bacteria, which only have teichoic acids. Accordingly, the *E. coli* spectrum in Figure 1 shows an intense peak at 1449 cm^−1^ (Band 28), due to phospholipids and LPS [19,29]. However, the amide II vibration (Band 32 at 1564 cm^−1^) displayed in the *S. epidermidis* spectrum was particularly intense, probably caused by the contributions from both the amine groups of the *N*-acetyl related vibrations at around 1530 cm^−1^ (Band 30) [29] (due to the peptidoglycans and to the glucosamine in the EPS), and from the vibrations as-signed to guanine and adenine (Band 32) [17].

### 3.2. Monitoring Bacteria Metabolism

Each population of bacteria was analyzed with respect to its metabolic evolution at fixed times of culture (i.e., 12, 24, and 48 h) in order to evaluate the behavior of the bands labelled in Table 1. We took into account the evolution over time of selected bands associated with the vibrations of the fundamental molecular components of the living bacteria, such as DNA, proteins, phospholipids, and polysaccharides.

**Figure 3:**
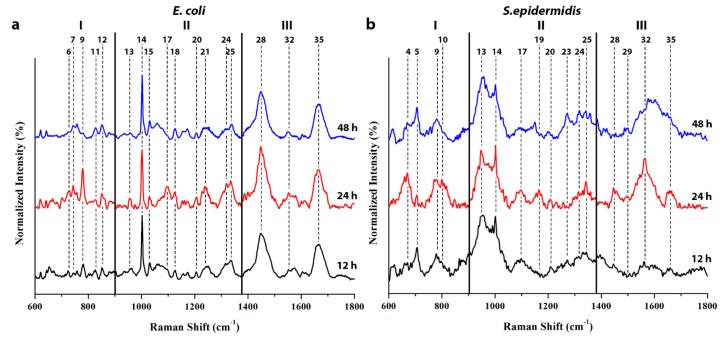
SERS spectra of *E. coli* (**a**) and *S. epidermidis* (**b**) in the 600 to 1800 cm^−1^ range, collected after 12, 24, and 48 h of culturing. The spectrum was divided into three distinct zones (labeled zones I to III), while the band frequencies were labeled according to Table 1.

Figure 3 shows the average SERS spectra collected at different times of the culture on living *E. coli* and *S. epidermidis*, respectively. The spectra have been divided into the same three main regions as previously described. The evolution of the bacteria population was monitored via fluorescence microscope imaging, too.

During the first 12 h of incubation, the bacterial population started to grow, with the density of bacteria incrementing exponentially and depleting the nutrients supply from the medium. Then, the growth stopped because of a lack of nutrients, reaching a maximum number of living bacteria at around 20 h of culture, entering in the stationary phase [29]. In this phase, the bacterial cells started to die by autolysis due to the lack of nutrients and the number of bacteria reached an equilibrium between the live and dead cells. Successively, the bacteria entered in a phase of senescence, where the number of death cells increasingly overcame that of the living ones, thus reducing the population [27]. Tests performed at 48 h can be associated with the beginning of this last phase.

The spectra shown in Figure 3 display tendencies that match the exponential/stationary/senescence phases of the growth curve. The *E. coli* spectrum (Figure 3a) displays a strong increment of eDNA related bands between 12 and 24 h of incubation, especially for the ring breathing vibration located at around 778 cm^−1^ in Zone I (Band 9); the origin of this spectral feature could be explained by the release and the accretion of eDNA from the bacterial cells into the biofilm matrix [24], as discussed above. Indeed, during the exponential phase, the eDNA, because of its poly-anionic nature [30], has been proven to have a key role in the formation of the extracellular matrix as a major structural component, increasing the inter-cellular cohesion and the exchange of genetic information [24,55]. Consistently, an increment of other nucleic acids’ related bands at 1096, and 1124, and ≅1560 cm^−1^ (Bands 17, 18, and 32, respectively), which have been previously assigned to the nitrogenous bases and to the phosphate groups vibrations, was observed. As stated before, the signal related to the phosphate groups shows a contribution both from nucleic acids and phospholipids, and indeed, it is more intense in the *E. coli* spectrum, probably for its higher amount of eDNA and for the components of its outer membrane.

Zones II and III display a similar time evolution; the vibrational modes assigned to the structural components of the cells, like the proteins in the amide I, II, and III region and the phospholipids, slightly increased. In particular, the amide III bands located at 1241 cm^−1^ and 1335 cm^−1^, and the amide II bands at 1553 cm^−1^ (Bands 21, 25, and 32, respectively) show the greatest intensity increment. After 48 h of culture, in the late stationary phase, the bacterial population starts to decrease, and this behavior can be clearly recognized by the reduction in the intensity of the bands related to nucleic acids, phospholipids, and proteins. As stated in previous works, the biofilm from the starvation phase (48 h) shows a substantial reduction of nucleic acids compared to protein [56,57], because of a concentration decrease in these specific components. Such results match with the data shown in Figure 3, where a dramatic reduction in the intensity is observed for the DNA related bands, particularly in Band 9, especially for *E. coli*, whereas the protein vibrational modes show a weaker signal reduction.

A similar time-pattern could be observed in Figure 3b for *S. epidermidis*. In the first 24 h, a strong increment of bands belonging to the DNA and structural components was noticed. Zone I in the SERS spectrum showed a significant increment in the intensity of the NAG related mode at 670 cm^−1^, which is one of the building blocks of the peptidoglycan layer, which constitutes the external wall of the Gram-positive bacteria, and of the EPS. This band can thus be used as a marker for the construction of new cell membranes, as well as for the above discussed biofilm. A slightly weaker increase was observed for the DNA band at 778 cm^−1^ (Band 9). A previous study by Campoccia et al. reports a positive correlation between the production of EPS and eDNA [30]; thus, such results match the tendency shown in the obtained results, in which the signals relative to EPS and DNA increase over time from 12 to 24 h, decreasing in the late stationary/senescence phase at 48 h.

At 24 h, Zones II and III exhibit an increase in the amide I, II, and III modes located at around 1657, 1563, and 1165–1342 cm^−1^, respectively (Figure 3), in comparison to the spectrum recorded at 12 h. Such a spectral enhancement indicates a rise in the amount of structural components, related to both the cells’ membrane and to the biofilm matrix. After 48 h of culture, the intensity of the highlighted bands decreased, suggesting that a variation in the bacteria metabolism occurred, similarly to what observed and reported above for *E. coli*.

**Figure 4:**
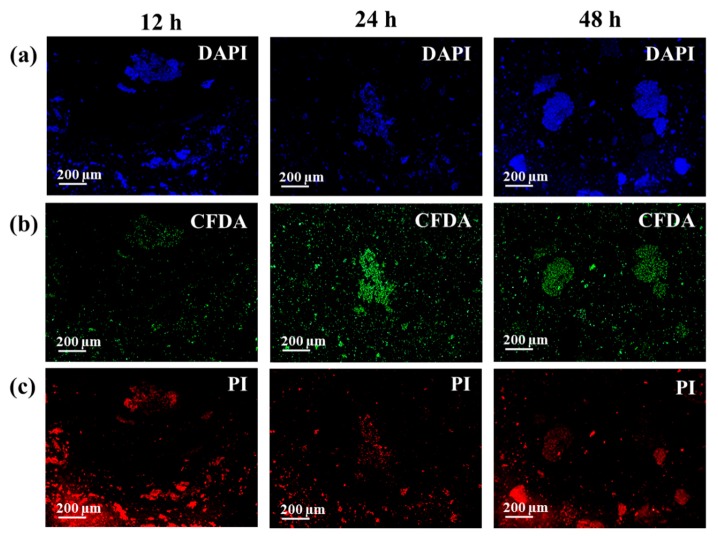
Fluorescence micrograph after propidium iodide (PI), 5(6)-carboxyfluorescein diacetate (CFDA), and 4′,6-diamidino-2-phenyilindole (DAPI) staining of *E. coli* cultured for 12, 24, and 48 h. Dead and living cells are labelled in red and green, respectively, while the nucleic acids are displayed in blue.

**Figure 5:**
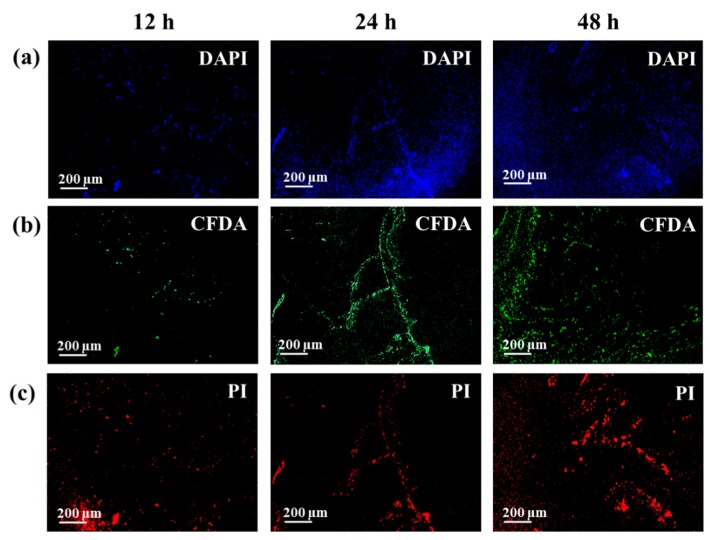
Fluorescence micrograph after PI, CFDA, and DAPI staining of *S. epidermidis* cultured for 12, 24, and 48 h. Dead and living cells are labelled in red and green, respectively, while the nucleic acids are displayed in blue.

A further validation of these trends is given by the fluorescence microscope images. The pictures of both bacteria, shown in Figure 4 and Figure 5, were taken at the selected time after staining with PI, CFDA, and DAPI, in order to evaluate the amount of dead bacteria, living bacteria, and nucleic acids, respectively. It can be seen in Figure 4b and Figure 5b that the number of living cells increases during the first 24 h, and then decreases after 48 h of incubation. Concurrently, the estimate of the number of dead cells shows an opposite behavior (Figure 4c and Figure 5c), meaning that, as stated before, after 48 h of culture, the bacteria start dying, reducing their population. The staining of both bacteria at 12 h also revealed a high number of PI positive cells, whereas the DAPI staining returned to a high background, especially with the *S. epidermidis* samples. It is worth noting that, even if a bacterial staining kit was exploited to perform the microscopy analysis, and the molecules used to label the different cells in their metabolic status are studied for their preferential interaction with a specific target (in particular, PI and DAPI, which bind nucleic acids), these markers can also bind other structural components. For instance, the usefulness of PI staining as a universal indicator for the viability of bacteria has been previously questioned; this compound tends to also label the viable cells of the Gram-negative and Gram-positive bacteria in the exponential growth phase [58]. Similarly, DAPI can also return a high fluorescence background and an erroneous enumeration of the positive cells, because of non-specific binding [59,60]. As a result of the presence of the EPS and the eDNA outside the bacterial cells, it is feasible that a certain amount of dye also binds these components during the staining. Then, even if the acquired images confirm the trends observed by the SERS analysis, they can be only used as qualitative tools. In any case, we tried to extrapolate the quantitative data from the images reported in Figure 4 and Figure 5, and also from those replicates not shown in the paper (*n* = 3). At the same time, we performed a viability assay (WST assay), in order to estimate the amount of living bacteria during this time. Both analyses showed trends that are coherent with the discussion of the data obtained by the SERS analysis, as reported in the Supplementary Material (Figure S2). Hence, all of the results obtained, especially the analysis of the vibrational features of the Raman spectra, pave the way for using pSi-based SERS substrates for the identification and metabolic analysis of Gram-negative and Gram-positive bacteria.

## 4. Conclusions

In this work, the potentiality of pSi-based SERS substrates was exploited in order to detect and discriminate different bacterial strains (*E. coli* and *S. epidermidis*). In comparison to the other techniques reported and discussed in the literature, the employment of these substrates for SERS analysis offers several advantages, such as the simplicity of fabrication, the low cost, and the possibility to develop a label-free bioassay, which allows for a minimal treatment of the samples. The latter feature guaranteed to preserve and to depict not only the bacteria itself, but also their biofilm matrix, for a complete characterization of the SERS spectra of both Gram-positive and Gram-negative bacteria, allowing for the identification of the main spectral differences between these strains. Moreover, the classification of the vibrational modes of each strain was utilized to discuss the bacterial growth dynamics. The biofilm matrix, along with the outer layer of the bacterial cells, has been proven to strongly contribute to the vibrational pattern of both bacteria strains, showing species related features, particularly concerning the bands assigned to the eDNA and to the presence of LPS, EPS, and peptidoglycans. In particular, the results show a strong increment of the intensity of the bands related to the structural components (proteins, lipids, phospholipids, and eDNA) during the first 24 h of culture, while a clear decrease of these bands was detected after 48 h of incubation, especially for the nucleic acids. It has been demonstrated that the time evolution of the highlighted bands shows a strong correlation with the biofilm matrix composition during bacterial growth. A further confirmation of this tendency is given by the fluorescence microscope images, which show an increase of the living cells during the first 24 h of culture, whereas after 48 h of incubation, a decrease is observed, concurrently with a rise of the amount of dead cells.

## Figures and Tables

**Table 1:** Surface enhanced Raman spectroscopy (SERS) assignments in Gram-negative and Gram-positive bacteria. NAG—*N*-acetylglucosamine; PDMS—polydimethylsiloxane.

	*E. coli* (cm^−1^)	*S. epidermidis* (cm^−1^)	Proposed Assignment	References
1	621		C–C twisting mode of phenylalanine	[23]
2	643	650	Guanine ring breathing	[17,19]
3		660	Guanine, thymine ring breathing	[23]
4	665	670	NAG	[27,29]
5	702	706	PDMS	[54]
6	725		Adenine ring breathing	[4,19,31,32]
7	744		B_1g_ heme vibration (cytochrome c)	[35,37,38,39,40]
8	755		Tryptophan ring breathing	[37,43]
9	778	780	DNA/RNA ring breathing (cytosine/thymine)	[17,27,29,31]
10		800	DNA/RNA ring breathing	[27,29]
11	826		ν_a_(O‒P‒O) str.	[19,44]
12	851	848	Thymine	[19,48]
13	955	950	ν(CH_3_) of proteins (a-helix)	[19]
14	1003	1002	Phenylalanine	[19,27,29,31,37]
15	1032	1030	Phenylalanine C–H in plane bending	[19]
16		1040	ν (CC) aromatic ring	[29]
17	1096	1097	ν_s_ (PO_2_)	[19,44]
18	1124		ν(PO_2_)	[19]
19		1165	Tyrosine, phenylalanine, amide III	[29]
20	1204	1208	Phenylalanine	[37]
21	1241	1230	ν(PO_2_^−^), amide III	[19]
22		1257	amide III	[19]
23		1277	PDMS	[54]
24	1319	1314	Guanine, CH_2_ twist (lipids)	[19,29]
25	1335	1342	Protein twisting (CH_2_ and CH_3_), ν(NH_2_) Adenine	[29,37]
26	1386		δ(CH_3_) symmetrical	[19]
27	1399		C‒O‒O^‒^ stretching in amino acids	[29]
28	1449	1446	Scissoring (fatty acids, phospholipids, and mono- and oligo-saccharides); CH_2_CH_3_ deformation	[19,29]
29		1495	δ (CH_2_)	[29]
30	1533	1529	Amide II of proteins, N-acetyl related bands (amide II)	[29]
31		1538	Amide II of proteins	[29]
32	1553	1564	Amide II of proteins, guanine/adenine (only *S. epidermidis*)	[17,19,29]
33	1579		Guanine, adenine, tryptophane (proteins)	[19]
34	1651	1648	Amide I of proteins (α—helix), cytosine/thymine	[17,19,29,46]
35	1667	1657	Amide I of proteins (random coils)	[46]
36	1694	1665	Amide I of proteins (β—sheet)	[46]

## Supplementary Materials

The following are available online at http://www.mdpi.com/2079-6374/8/4/131/s1, Figure S1: Comparison between Raman and SERS spectra of *E. coli* in the 600–1800 cm^−1^ range, collected after 24 h of culturing. Figure S2: Amount of living and dead bacterial cells of *E. coli* and *S. epidermidis* obtained by the image analysis and by the WST8 viability assay.
