# Supplementary material for: Label-Free SERS Discrimination and In Situ Analysis of Life Cycle in Escherichia coli and Staphylococcus epidermidis
Source: Biosensors (Basel). 2018 Dec 15;8(4):131. doi: 10.3390/bios8040131 (PMC6315751; doi:10.3390/bios8040131)
Supplement: Supplementary file 1 [file biosensors-08-00131-s001.pdf]

Supplementary Materials

Label-Free SERS Discrimination and In Situ Analysis of Life Cycle in *Escherichia coli* and *Staphylococcus epidermidis*

Niccolò Paccotti, Francesco Boschetto, Satoshi Horiguchi, Elia Marin, Alessandro Chiadò, Chiara Novara, Francesco Geobaldo, Fabrizio Giorgis and Giuseppe Pezzotti

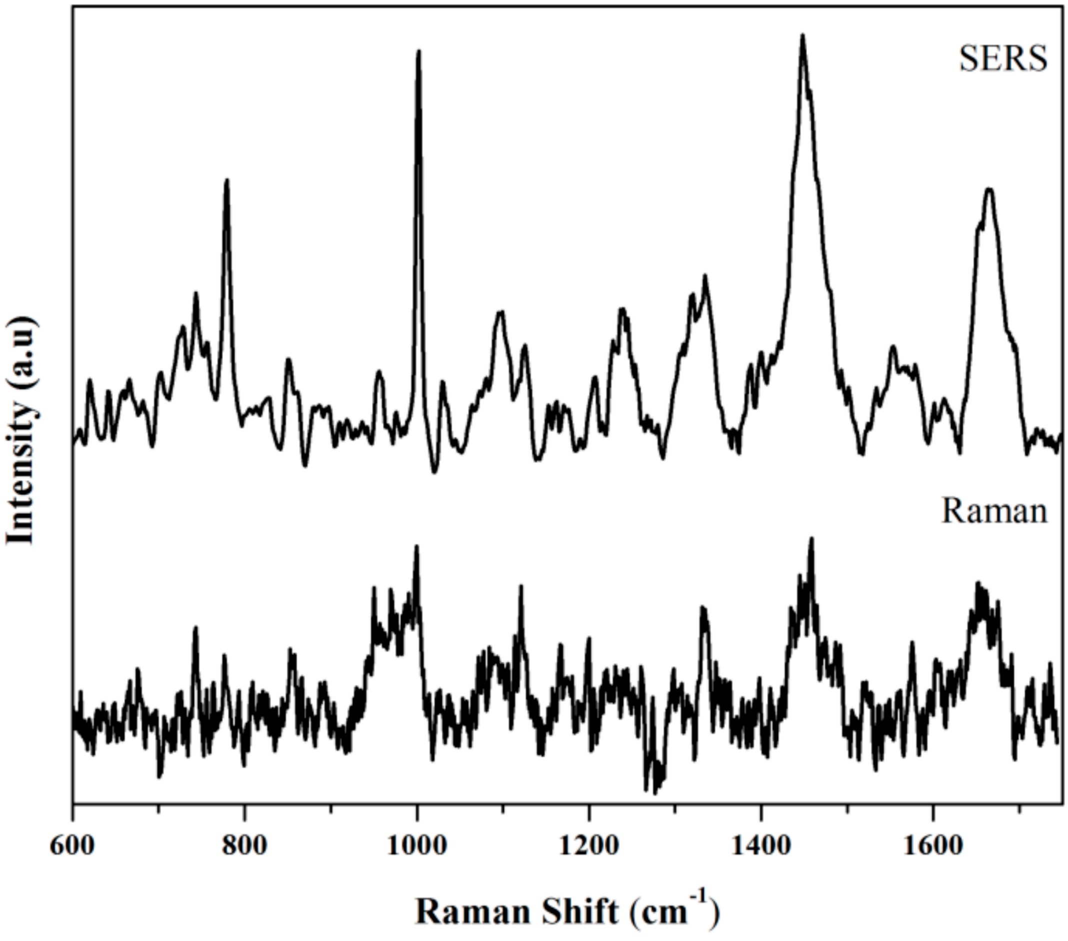

Figure S1. Comparison between Raman and SERS spectra of *E. coli* in the 600–1800 cm^{-1} range, collected after 24 h of culturing.

The comparison of the SERS and Raman spectra of *E. coli*, reported in Figure S1 as an example, evidences a specific enhancement of the vibrational modes assigned to the outer membrane and to the biofilm matrix, when the measurements are acquired on the silver coated pSi-PDMS membranes, probably due to the close proximity of the extracellular and membrane material to the plasmonic nanoparticles. This effect is clearly noticeable for the main eDNA bands at 725 cm^{-1}, 788 cm^{-1} and 1319 cm^{-1}, but a change in the shape of the 1450 cm^{-1} band could also witness an increased contribution of the phospholipids on the outer membrane of *E. coli* to the SERS spectrum in comparison to the Raman

Biosensors **2018**, *8*, 131; doi:10.3390/bios8040131

one. SERS analysis therefore enables the sensitive detection of those features that better characterize the different bacterial strains, allowing their discrimination.

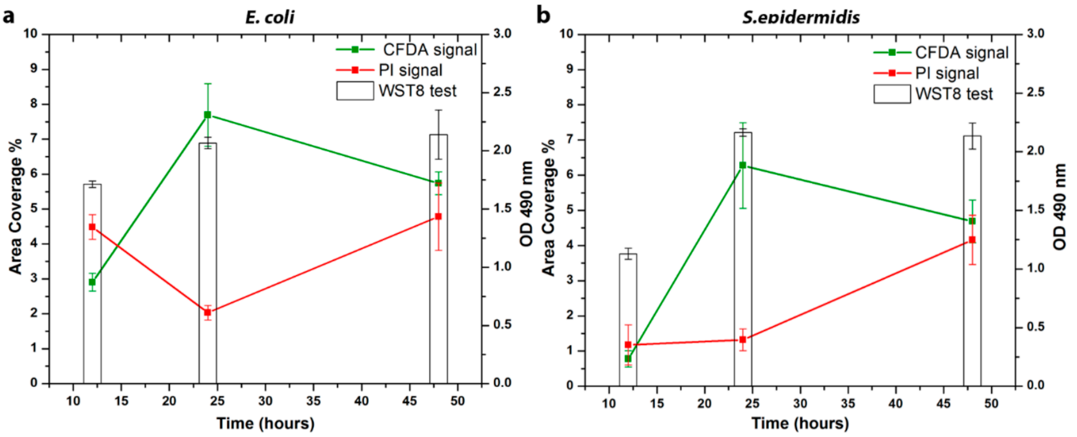

Figure S2. Amount of living and dead bacterial cells of *E. coli* and *S. epidermidis* estimated by the image analysis (Area Coverage % of the 5(6)-carboxyfluorescein diacetate (CFDA, green) and propidium iodide (PI, red) signals) and by the optical density (OD) recorded at 490 nm as readout of the Microbial Viability Assay Kit-WST8.

Even if the fluorescence images can be only used as qualitative tools, we tried to extrapolate quantitative data from the images reported in Figures 4 and 5 and also from those replicates not shown in the paper (n = 3). At the same time, we performed a viability assay (Microbial Viability Assay Kit-WST8), in order to estimate the number of living bacteria during time. Both analysis return trends that are coherent with the discussion of the data obtained by SERS analysis. In particular, the amount of living bacteria (CFDA, signal in green) is increasing at 24 h, in comparison to 12 h. This amount is instead reduced after 48 h of culture. The same trend can be recorded by the WST8 assay, which highlighted the higher amount of living bacteria at 24 h, in comparison to 12 h, as well as the equilibrium between live and dead cells during the late stationary phase at 48 h. Indeed, the amount of dead cells (PI, signal in red) is increasing during time, especially if the values at 24 and 48 h are compared. The high signal of PI recorded for *E. coli* at 12 h is probably due to the non-specific binding of PI, which increase the background noise of the acquired images.
